# Establishing evidence to inform culturally competent mental health services: A mixed methods study protocol

**DOI:** 10.1371/journal.pone.0282445

**Published:** 2023-03-14

**Authors:** Ge Yu, Maria Panagioti, Eugene Y. H. Tang, Damian Robinson, Nusrat Husain, Reena Lasrado, Karina Lovell, Eileen Kaner, Yu Fu

**Affiliations:** 1 Population Health Sciences Institute, Newcastle University, Newcastle, United Kingdom; 2 NIHR Applied Research Collaborative North East and North Cumbria, Cumbria, Northumberland and Tyne and Wear NHS Foundation Trust, Gosforth, Newcastle upon Tyne, United Kingdom; 3 Centre for Primary Care and Health Services Research, University of Manchester, Manchester, United Kingdom; 4 North East and North Cumbria, Cumbria, Northumberland and Tyne and Wear NHS Foundation Trust, Gosforth, Newcastle upon Tyne, United Kingdom; 5 Mersey Care NHS Foundation Trust, Prescot, United Kingdom; 6 Division of Nursing, Midwifery & Social Work, University of Manchester, Manchester, United Kingdom; 7 NIHR Applied Research Collaborative Greater Manchester, Manchester University NHS Foundation Trust, Manchester, United Kingdom; Public Library of Science, UNITED STATES

## Abstract

**Background:**

COVID-19 has exacerbated the significant and longstanding mental health inequalities for ethnic minorities, who were less likely to access mental health support in primary care but more likely to end up in crisis care compared to the majority ethnic group. Services were poorly offered and accessed to respond to the increased mental health challenges.

**Aim:**

To 1) establish evidence on which changes to mental health services provided in response to COVID-19 are beneficial for ethnic minorities who experience mental health difficulties in the North of England, and 2) to inform what and how culturally competent mental health services should be routinely provided.

**Methods:**

A mixed methods approach comprising 1) a rapid review to map services and models of care designed or adjusted for mental health during the pandemic, 2) an observational study of retrospective routine data to assess changes to mental health services and associated outcomes, 3) qualitative interviews to understand experiences of seeking care and factors associated with high-quality service provision, and 4) a Delphi study to establish consensus on key features of culturally competent services. From the selected areas in the North of England, adults from ethnic minorities who experience mental health difficulties will be identified from the primary, community and secondary care services and local ethnic minority communities.

**Discussion:**

This study will identify ways to tackle health inequalities and contribute to mental health service recovery post pandemic by providing practice recommendations on equitable and effective services to ensure culturally competent and high-quality care. A list of services and features on what and how mental health services will be provided. Working with study collaborators and public and patient involvement partners, the study findings will be widely disseminated through presentations, conferences and publications and will inform the subsequent funding application for intervention development and evaluation.

## Introduction

It is recognised that as well as physical effects, a COVID-19 diagnosis has adverse mental health effects both in previously healthy people and those with pre-existing mental health disorders [1]. About 1 in 5 people with COVID-19 have experienced poorer mental health within 90 days of diagnosis [2]. Risk for developing a mental health problem in patients with COVID for the first time is twice as likely as those without, and risk can be three times higher for developing dementia amongst over 65s with COVID [3]. People with existing psychiatric diagnoses have also reported increased symptoms and poorer access to services and support leading to relapse and suicidal behaviour [1]. At the same time, they are more likely to contract COVID-19 than people with no history of poor mental health [3].

COVID-19 has had a disproportionate impact on people from ethnic minorities in the UK including overall numbers of positive cases, relative numbers of critical care admissions and deaths [4–6]. Public Health England found that people from Black ethnic groups had the highest age-standardised COVID-19 diagnosis rates whereas White ethnic groups had the lowest [7]and that people of Bangladeshi ethnicity were twice more likely to experience a COVID-related death than White British people [5]. This differential risk by ethnicity is likely to be multifactorial such as poorer baseline health, deprivation, having jobs exposing them to higher risk, unemployment and financial stress, and cultural and language differences placing additional barriers to accessing services [7].

The percentage of adults and older who reported experiencing a significant level of psychological distress has increased during the pandemic [8]. Inequalities in mental health have been further exacerbated for ethnic minority communities, especially as there were already significant and longstanding mental health inequalities for them prior to COVID-19 [9, 10]. Higher levels of anxiety and depression and worse mental health were reported in ethnic minorities across the pandemic than White ethnic groups [11]. Recent research also revealed persistent ethnic-gender specific changes in mental health between pre and during the COVID-19 pandemic and reported that Bangladeshi, Indian and Pakistani males experienced the highest average increase in mental distress compared with White British males [12]. The average mental health status was worse in people from an ethnic minority background over time in the North compared to the rest of England [13, 14]. These inequalities have been exacerbated and deepened in several ways by the pandemic due to lockdowns, constraints of quarantine and disrupted or closure of some usual clinical care and supporting services for people with mental health problems. In addition, face to face appointments were replaced with remote consultations, but they were not always accessible for many groups of vulnerable population [15], including those experiencing material deprivation and/or with low confidence in using digital technology.

Evidence showed that ethnic minority service users received less and worse mental health care and/or poorer access to services. A review [16] commissioned by NHS England found that minority ethnic communities were less likely to access mental health support in primary care and were more likely to end up in crisis care. Compared to white people, they were 40% more likely to access mental health services through the criminal justice system. There were also substantial reductions in first diagnoses of anxiety and depression disorders compared with expected rates during the lockdown, and fewer people sought mental health support from their GP or hospital, particularly for those from disadvantaged groups. This suggested that existing inequalities in mental health grew wider during the pandemic [17]. In addition, the caseload of patients accessing secondary care (urgent and emergency cases) increased [18]. This could mean that patients were unable to access support until their condition had worsened [15]. The health of ethnic minorities has further been negatively impacted by the lack of accessible interpreting services resulting in delays or avoidance of seeking help due to fear of racist treatment [19]. Consequently, care and services provided were not felt adequate to respond to the demand observed for increased mental health problems [20]. For those who had access to support services including medical and social services, they did not feel that the support they were offered was effective and satisfied their needs. Some believed that the government focused on more morbidity and hospitalisation risks from COVID-19 but ignored the effects of lockdown on population mental health [21]. Both national and international organisations advocate for integration of mental health and psychosocial support into the COVID-19 response [22]. To act promptly and proactively in response to patients’ individual needs, researchers are suggested to analyse data covering not only hospital admissions but also data from primary care, linking information on mental health, COVID-19 and ethnicity [23]. This provides an opportunity to rethink conventional approaches to service planning and ways for greater inclusion of ethnic minorities to reduce inequalities and improve the scale, effectiveness and quality of mental health services [24]. Therefore it is vital to learn from service changes and their consequences for ethnic minorities in order to inform policy solutions for integrated service recovery and plan for culturally competent services.

The overall aim of this study is to 1) establish evidence on which changes to mental health services provided in response to COVID-19 are beneficial for ethnic minorities who experience mental health difficulties in the North of England, and 2) to inform what and how culturally competent mental health services should be routinely provided to contribute to mental health service recovery.

The following objectives underpin the work packages:

1. Map health services and models of care designed or adopted for mental health conditions during the pandemic and their impacts.
2. Identify and describe changes in mental health service utilisation and consequences for ethnic minorities between four-time points: pre-lockdown (03.2019–03.2020), full lockdown (03–07.2020), post lockdown (05.2020–02.2021) and end of restrictions (07.2021–03.2022).
3. Understand the experience of seeking mental health support during COVID-19 and identify factors that enabled the provision of high-quality services and patient engagement.
4. Synthesise evidence generated and establish consensus across patients and professionals on resetting acceptable, effective and culturally competent mental health services.

## Materials and methods

### Design

A mixed methods approach with sequential design will be employed, comprising a rapid review and synthesis of current evidence (Workpackage (WP1)), quantitative analysis of retrospective routinely collected National Health Service (NHS) data (WP2), qualitative interviews to understand experiences of patients from ethnic minorities and factors facilitating high-quality service provision during the pandemic (WP3), and a Delphi survey of patients, professionals and commissioners to establish consensus on recommendations for culturally competent services and core services post pandemic (WP4).

### Ethics

Ethical approvals was granted by the Health Research Authority Ethics Committee (22/YH/0129) to conduct WP2 using routine data and to process confidential patient information without consent (22/CAG/0092). Data processing and sharing agreements will be in place. All data will be pseudonymised with a common pseudonym before being sent to Newcastle University via secure file transfer.

Ethical approval was also granted by the Health Research Authority Ethics Committee (22/WS/0164) to conduct WP3 and WP4. Written and electronic consent will be sought from participants and stored securely on the University’s server.

### Setting

Data collection (WP2-4) will be undertaken in the North East and North Cumbria (NENC) and Greater Manchester (GM), England where mental health prevalences are high but underserved by mental health research delivery.

### Patient and public involvement (PPI)

This study has been designed with people with lived experience and the Voluntary Community and Social Enterprise (VCSE) sectors, who closely link to and provide support to their local ethnic minority communities. A short survey was distributed to 16 members (all from ethnic minority backgrounds) of one of the VCSE sectors and results indicated that the majority (n = 12) suffered from some degree of mental health problems and a half from moderate and severe mental health illness. All agreed that mental health is an area of unmet need with exacerbated inequalities due to the pandemic, and suggested “*translation services*” to be available for those who have language barriers, as they are “*difficult to engage*” groups.

A group of 2–4 PPI partners has been convened who will continue to help develop and review study materials and dissemination plans. They have been ensured that a flexible approach will be adopted and we plan to invite them to define their activities at the outset and that we accept a responsibility to value and use their contributions. The VCSE sectors will support participants’ recruitment and dissemination to make sure the study results are acceptable and accessible to people from a range of backgrounds.

### WP1

The protocol for a rapid review was published [25] to understand the changes in mental health services during the pandemic and summarise the impact of these changes on reported health outcomes of people with mental health conditions.

### WP2

WP2 aims to quantify changes to mental health utilisation due to the pandemic and health outcomes, identify the specific factors at both individual and general practice levels contributing to these changes, and estimate the association between mental health service utilisation and patients’ health outcomes of ethnic minority groups.

#### Sampling

Currently registered patients (18+) diagnosed with anxiety and depression before 23^rd^ March 2019 who were referred or self-referred to NHS-funded secondary mental health services or Improving Access to Psychological Therapies (IAPT) between 23^rd^ March 2019 to 22^nd^ March 2020 will be included. Patients who had mental health conditions without having been referred or self-referred before 23rd March 2020 or who have moved out of the NENC region will be excluded.

A total enumeration approach will be used to include all eligible participants from six selected practices located in three areas with high ethnic minority populations, as identified by the 2021 UK Census. The Quality and Outcomes Framework (QOF2020-21) indicated an estimated 12.3% of the national population with depression recorded on their practice register. Therefore, a total sample of 1,328 non-white adults is estimated in contact with NHS-funded secondary mental health services in the six practices. As a general assessment of power to detect changes in waiting time comparing before and after the COVID-19 disruption to services, power for the simplistic situation can be estimated assuming a paired t-test is to be used to detect changes in waiting time. A total of 1,328 participants would allow 95% power at the two-sided 5% significance level to detect a true difference of 0.08 standard deviations in waiting time (days). This sample size calculation is a guide, as in reality the data will consist of repeated measurements of the participants and more appropriate methods will be applied for the analysis which takes account of the repeated measurements and nested nature of the data.

#### Data source

The primary care data source will be the local primary care electronic patient records that contain anonymised patient level data from the six identified general practices. In addition, we will link the primary care data with the Hospital Episode Statistics (HES) dataset (i.e., admitted patient care, outpatients, and Emergency Care Data Set), Mental Health Services Data Set (MHSDS), Improving Access to Psychological Therapies (IAPT) data set, and Community Service Data Set (CSDS). The study will also utilise the GP practice-level data from the Fingertips [26], which is a publicly accessible web tool (https://fingertips.phe.org.uk/) maintained by Public Health England.

All the data sources will be requested, processed and linked by North England Commissioning Support Unit (NECS) using unique identifiers. The linked data will be collected for three years, from 23^rd^ March 2019 to 22^nd^ March 2022.

#### Outcome measures

Changes to mental health services utilisation in response to COVID-19 and their impact on health outcomes will be identified from the rapid review of the literature in WP1 [25]. Mental health services will include referrals to new services, access to mental health services, modes of contact with services, pattern of mental health service utilisation, treatment adherence/completion, and prescription of antidepressants.

The primary health outcomes will include mental health, physical health, self-harming, psychotropic medication, common mental disorder symptoms, morbidity, mortality, time to first readmission, and use of A&E or A&E readmissions within 30 days. In addition, the study may investigate selected outcomes for which there is unknown evidence of an association with service utilisation. Individual-level socio-demographic and clinical characteristics and GP practice-level characteristics will be used to control for the potential influence of confounders or mediators on the relationships between the pandemic, mental health service utilisation and related health outcomes.

#### Data analysis

All statistical analyses will take into account any relevant confounding variables. The study will examine the entire sample as well as specific subgroups based on factors such as age, gender, ethnicity, and socioeconomic status. All calculations will be performed using the latest version of Stata software (version 17.0) [27].

To understand the demographic and clinical background of the sample, a descriptive approach will be used to analyse characteristics such as age, gender, diagnosis and prescriptions, in the year leading up to the first lockdown. These characteristics will be compared using appropriate methods including t-test, rank-sum, and chi-square test, and consider a p-value of less than 0.05 as statistically significant. If any imbalances in healthcare utilisation are found to be related to specific variables, and these variables have a correlation of at least 0.3 with outcomes, these variables will be included as control factors in regression analyses. Advanced statistical techniques, multi-level regression analysis, will be used to analyse the utilisation of mental health services among our sample throughout the different phases of the lockdown. This method takes into account both individual-level and practice-level factors that may influence utilisation. A three-level model will be constructed that considers the hierarchical structure of the data, where patients are grouped within practices and practices are grouped within time periods. We assume that lockdown regulations are consistent across different regions, and that there are variations among practices, which will be accounted for in the analysis to provide an accurate estimate of the model parameters.

To investigate the relationship between changes in healthcare utilisation and health outcomes, latent growth models will be conducted within a structural equation modeling framework. This method examines how the utilisation of healthcare services changes over time, and how it relates to changes in health outcomes. The analysis will be broken down into different time periods, and we will fit separate growth curves for each period. Additionally, how two or more outcome variables change over time will be examined with each other, for example, the change in waiting time and the change in health outcomes. We will also take into account individual-level and practice-level characteristics as potential predictors of health outcomes.

For our primary analyses, we will not use imputation methods for missing data if it is determined that the missing values are completely random (MCAR). However, if it is found that the missing data is not MCAR, we will use multiple imputation by chained equations to create 10 additional datasets under the assumption that the missing data is random (MAR). Known predictors and stratification variables will be used to estimate the missing values. The results will be presented as a pooled summary from the 10 datasets. It’s possible that during the research, missing data or variables may be discovered, If so, any deviations from the original plan will be reported and implications on the research and conclusions will be discussed. Additionally, sensitivity analyses may also be conducted to assess the potential impact of missing data on the study population and conclusions.

### WP3

WP3 aims to understand which and how services to patients with mental health problems changed during COVID-19, difficulties experienced and solutions provided, and factors that facilitated or hampered the quality of services and patient engagement.

#### Participants and recruitment

20–30 service users (18+) from different ethnic minority groups with mental health problems diagnosed pre COVID first lockdown (March 2019) will be targeted. Patients under palliative care will be excluded.

Eligible participants will be recruited from both GP practices and clinical services including Cumbria, Northumberland Tyne and Wear NHS Trust (CNTW) clinical services, Northern Care Alliance Trust, Greater Manchester Mental Health NHS Foundation Trust, Bolton NHS Foundation Trust, Mersey Care NHS Foundation Trust, Community Learning Disability and Integrated teams through Local Care Organisation (Local Authorities). Study materials and social media (Twitter, Facebook) in multi-languages will be used to support the recruitment process.

A purposive sampling will be used to recruit service users taking into account age, sex, ethnicity (targetting ethnic minority communities), mental health severity and comorbidities. Recruitment will continue until sufficient data is collected to ensure a broad range of experiences.

#### Data collection

With participants’ informed consent, data will be collected using semi-structured individual interviews which will be undertaken face to face or on an appropriate virtual online platform, such as Microsoft Teams or Zoom according to participants’ preferences. Interpreters can be arranged if required.

An interview schedule has been co-developed with the PPI partners and the interview will be guided by the participants whatever possible. Areas of questions to be explored include:

- Demographics and overview of mental health conditions
- Expectations and experience of mental health services pre COVID-19, barriers and facilitators to quality of the services provided
- Mental health services offered, accessed and delivered during the pandemic, barriers and facilitators to quality of the services provided
- Association between ethnicity and mental health services provided including inequalities experienced and cultural needs

#### Data analysis

Interviews will be recorded with informed consent. Recorded interviews will be transcribed by a professional transcription company. Participants will be given pseudonyms to preserve their anonymity and any identifiable information remaining will be removed.

Framework analysis [28] will be used to define and categorise data into themes and subthemes. A framework matrix will also be generated summarising the data from each transcript. An analytical framework will evolve as the coding process progresses and themes emerge. A sample of three interviews will be double-coded independently by two researchers as a validity check. The research project team and PPI partners will discuss emerging analyses to ensure rigour, discuss differences and agree upon the analytical framework. Nvivo will be used to manage and analyse the data [28].

### WP4

WP4 aims to establish a consensus between ethnic minorities (with mental health conditions and/or their carers), professionals and commissioners on what acceptable and effective core services and how they should be routinely provided to ensure culturally competent care.

#### Participants and recruitment

An expert panel will be established involving ethnic minorities (over 18) with mental health conditions (both diagnosed and self-reported), their families or carers, professionals and commissioners from both regions. They will be included if they access the Internet and email. Participants involved in the qualitative interviews will also be invited to join the panel if they consented to agree to be contacted for future relevant studies.

There are no established guidelines on the optimal Delphi study panel size [29]. A target of 24 to 60 panellists (8–20 per group) will be set to ensure key stakeholders are sufficiently represented and the panel remained manageable. Panellists will be selected using stratified purposive sampling of a minimum number of ethnic minorities, professionals and commissioners from key groups, availability sampling and snowball sampling of ethnic minorities and professionals [30–32]. Panellists who completed the survey will be invited (only if they consented) to attend a consensus workshop with up to 10 members to determine recommendation for service provision and key factors/characteristics that enabled high-quality mental health service.

Ethnic minorities with mental health problems will be recruited using the same strategy described for WP3. In addition, local collaborative communities will support the recruitment of those who have experience with mental health services. Health professionals will be recruited from the clinical services of CNTW, Northern Care Alliance Trust, Greater Manchester Mental Health NHS Foundation Trust, Bolton NHS Foundation Trust, Mersey Care NHS Foundation Trust and the Mental Health Special Interest Group supported by the NIHR Applied Research Collaboration for the NENC. Commissioners will be recruited from the NENC Integrated Care Board (ICB) and Integrated Care System Mental Health Workstream, NHS Cheshire and Merseyside ICB, NHS Greater Manchester ICB, and NHS Lancashire and South Cumbria ICB. To maximise the diversity of the panel a range of social media sources (Twitter, Facebook) will be drawn targeting health professionals who are involved in the organisation and implementation of services and care for mental health patients.

#### Data collection

*Delphi study*. Evidence generated from WP1-3 will be summarised and integrated and reviewed by the research team and discussed with the PPI partners. This will inform the content of a two-round online modified Delphi study. Both surveys will be hosted using the Online Surveys tool and administrated via email. A consent statement will be included on each survey’s introductory page. Reminders will be provided via email to help maximise response rates [33]. All individuals who completed Round 1 will be subsequently emailed links to Round 2.

In Round 1, an open-ended survey will be sent by email to the panel showing the list of services and features of service provision identified as effective and acceptable. Panellists will be asked to rate each item on a 9-point Likert scale from ‘Not at all important’ to ‘Very important’. Free-text options will be included at the end of each recommendation section to allow panellists to suggest additional items. Round 1 will also include questions on panellists’ characteristics. Separate sets of questions will be included for panellists with mental health problems (focused on their socio-demographic and clinical characteristics) and professional panellists (focused on their workplace, role and experience).

Round 2 will follow the same format as Round 1 with inclusion of all the Round 1 items accompanied by three charts showing panellists’ importance ratings for each item in Round 1. They will be provided with the opportunity to reconsider their judgements and refine their responses if necessary. No free text options will be included in Round 2 to minimise panellist and research burden.

*Consensus workshop*. Clear objectives, agenda, and materials (handouts, presentation slides, a summary of the survey outcomes) will be prepared and shared prior to the workshop (delivered virtually by the research team for up to 60 min) so that members and PPI partners are aware of what is to be discussed. The workshop will be facilitated by both researchers and PPI partners. With participants’ consent, this workshop will be recorded.

#### Data analysis

*Delphi study*. There are no established guidelines on how to define consensus in Delphi studies, per cent agreement is frequently used [34] and 70% is a commonly specified threshold [35]. Consensus is therefore provisionally defined as at least 70% of respondents rating an item as ‘Important’ or ‘Very important’. This will be verified using the interquartile range where ≤ 1 is defined as having achieved consensus [36]. Responses will be analysed for all panellists considered together and for patient and professional panellists separately. All items that reached consensus in Round 2 amongst all respondents considered together will be included in the final set of recommendations.

The Round 1 free-text responses will be analysed using directed content analysis [37, 38]. Each recommendation section will be considered a main category and each recommendation item will be considered a potential sub-category. The free-text responses will be inductively coded. Where possible, codes will be included within the pre-specified sub-categories which will be grouped into generic categories. All inductively generated sub-categories will be considered potential new items of inclusion in Round 2. If Round 2 takes longer than 30 min to complete, in which case only new items suggested by more than a threshold percentage of panellists will be included. This approach is chosen to help ensure that potentially important items are not omitted from consideration, whilst also ensuring the time burden for panellists remains manageable.

*Consensus workshop*. The automatic transcription generated by the online meeting software will be pseudonymised and analysed using content analysis. The agreed recommendations will be interpreted and reviewed in light of emerging evidence, with support from the PPI partners.

## Discussion

This project is expected to have an immediate impact on the care of patients with mental health conditions by increasing awareness of the impacts associated with service changes and factors enabling high-quality services, for both affected individuals and their service providers. Increased awareness in primary care may lead to proactive identification and improved care for these patients who are at risk through tailored communication and culturally sensitive approaches. Mapping changes in mental health services throughout the region will also identify opportunities for immediate care pathway improvements that are not dependent upon commissioning.

Through examining patients’ pathways as well as lived experience, service received and service needed will be identified, leading to the recognition of opportunities for service improvement. Subsequent research applications will be made to support patient-centred evidence-based service development, evaluation and implementation.

### Study limitation

With routine data, it focuses on the North East which might result in generalisability limitation however the North East is underrepresented in other national datasets such as CPRD [39]. The quality (accuracy and completeness) of data recorded will influence the reliability of the findings. Associations might be over or underestimated due to unobserved confounders or systematic bases in measurement errors. With interviews, not all ethnic minorities may be recruited. Also, the involvement of interpreters in some of the interviews might impact the dynamics and accuracy including tone and emphasis. In addition, all panellists will be required to be able to use/access email and communicate in English for the Delphi study, which may not be fully representative of all patients in this group.

### Dissemination

A study website will be set up for ongoing dissemination to all audiences. Plain language summaries and short videos will be co-produced with the PPI partners describing study findings on social media and other support organisations.

#### Ethnic minorities with mental health conditions

Participants will help us disseminate the research. The dissemination to and with ethnic minorities will be ongoing throughout the project and we will present findings at their communities and forums identified by participants and the VCSE sectors as having a strong impact through presentations at their network meetings, events and the community forum. Study information will be shared through:

- National Institute for Health and Care Research (NIHR) Three Research Schools
- NIHR Applied Research Collaboration Mental Health Implementation Network
- Newcastle City Council and Gateshead Public Health teams
- Connected Voice bulletins (~3000 contacts), social media and quarterly magazine
- The National Council for Voluntary Organisations
- Voluntary Sector North West
- Greater Manchester Centre for Voluntary Organisation
- Manchester’s local voluntary & community sector support organisation
- Manchester BME Network
- Manchester City Council Neighbourhood

#### Health professionals

We will disseminate to regional and national healthcare audiences locally through the linked NIHR infrastructure and via clinical collaborators, NENC ICS Mental Health Workstream, Mental Health Clinical Network and GM Health and Social Care Partnership (HSCP). A summary will be shared with the involved practices and clinical services of the mental health Trusts, and with policymakers via the NIHR Policy Research Unit networks.

#### Academics and researchers

In addition to our report to NIHR, we will publish 2 high-quality papers from the WPs1-4. We will also target the Society for Academic Primary Care Annual Conference attended by not only health professionals but also health researchers and academics.
