# Supplementary material for: Establishing evidence to inform culturally competent mental health services: A mixed methods study protocol
Source: PLoS One. 2023 Mar 14;18(3):e0282445. doi: 10.1371/journal.pone.0282445 (PMC10013880; doi:10.1371/journal.pone.0282445)
Supplement: Peer review history

# Editor report: Decision Letter 0

**DOI:** 10.1371/journal.pone.0282445.r001

**Authors:** Avanti Dey

15 Feb 2023

Establishing evidence to inform culturally competent mental health services: a mixed methods study protocol

PONE-D-23-03010

Dear Dr. Yu,

We’re pleased to inform you that your manuscript has been judged scientifically suitable for publication and will be formally accepted for publication once it meets all outstanding technical requirements.

Within one week, you’ll receive an e-mail detailing the required amendments. When these have been addressed, you’ll receive a formal acceptance letter and your manuscript will be scheduled for publication.

An invoice for payment will follow shortly after the formal acceptance. To ensure an efficient process, please log into Editorial Manager at http://www.editorialmanager.com/pone/, click the 'Update My Information' link at the top of the page, and double check that your user information is up-to-date. If you have any billing related questions, please contact our Author Billing department directly at authorbilling@plos.org.

If your institution or institutions have a press office, please notify them about your upcoming paper to help maximize its impact. If they’ll be preparing press materials, please inform our press team as soon as possible -- no later than 48 hours after receiving the formal acceptance. Your manuscript will remain under strict press embargo until 2 pm Eastern Time on the date of publication. For more information, please contact onepress@plos.org.

Kind regards,

Avanti Dey, PhD

Staff Editor

PLOS ONE

Journal Requirements:

1. Please note that in order to use the direct billing option the corresponding author must be affiliated with the chosen institute. Please respond by return e-mail so that we can amend your submission or remove this option. We can make any changes on your behalf.

Additional Editor Comments:

We have assessed your submission as being eligible for an expedited Accept decision based on the quality of the submission and documentation provided. Thank you for submitting your work to PLOS ONE!

​

# Editor report: Acceptance letter

**DOI:** 10.1371/journal.pone.0282445.r002

**Authors:** Avanti Dey

6 Mar 2023

PONE-D-23-03010 

Establishing evidence to inform culturally competent mental health services: a mixed methods study protocol 

Dear Dr. Yu:

I'm pleased to inform you that your manuscript has been deemed suitable for publication in PLOS ONE. Congratulations! Your manuscript is now with our production department. 

If your institution or institutions have a press office, please let them know about your upcoming paper now to help maximize its impact. If they'll be preparing press materials, please inform our press team within the next 48 hours. Your manuscript will remain under strict press embargo until 2 pm Eastern Time on the date of publication. For more information please contact onepress@plos.org.

If we can help with anything else, please email us at plosone@plos.org. 

Thank you for submitting your work to PLOS ONE and supporting open access. 

Kind regards, 

PLOS ONE Editorial Office Staff

on behalf of

Dr. Avanti Dey 

Staff Editor

PLOS ONE
